# Supplementary material for: High-Efficiency Extraction of Pantoea alhagi Exopolysaccharides Driven by pH-Related Changes in the Envelope Structure
Source: Molecules. 2022 Oct 25;27(21):7209. doi: 10.3390/molecules27217209 (PMC9657395; doi:10.3390/molecules27217209)
Supplement: Supplementary file 1 [file molecules-27-07209-s001.zip › molecules-1959199-supplementary.pdf]

High-efficiency extraction of *Pantoea alhagi* exopolysaccharides driven by pH-related changes in the envelope structure

Yuhang Ma[1,†], Liang Sun[1,†], Rui Wang[1,], Yian Gu[1],Hong Xu[1,*], Peng Lei[1*]

[1] State Key Laboratory of Materials-Oriented Chemical Engineering, College of Food Science and Light Industry, Nanjing Tech University, Nanjing 211816, China

***Corresponding author**

Hong Xu; Nanjing Tech University; Tel/Fax: +86-25-58139433; E-mail address: xuh@njtech.edu.cn;

Peng Lei; Nanjing Tech University; Tel/Fax: +86-25-58139433; E-mail address: lei-peng@njtech.edu.cn;

[†] These authors contributed equally to this work.

The following are Supplementary information to this article :

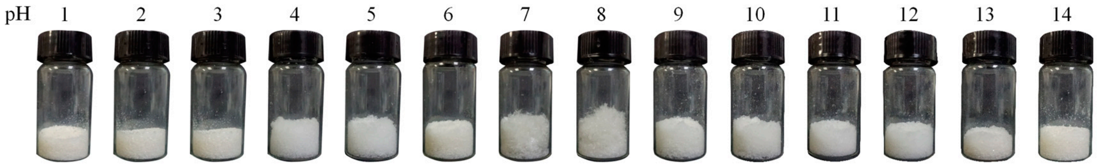

Figure S1. Dried PAPS obtained by different treatments.

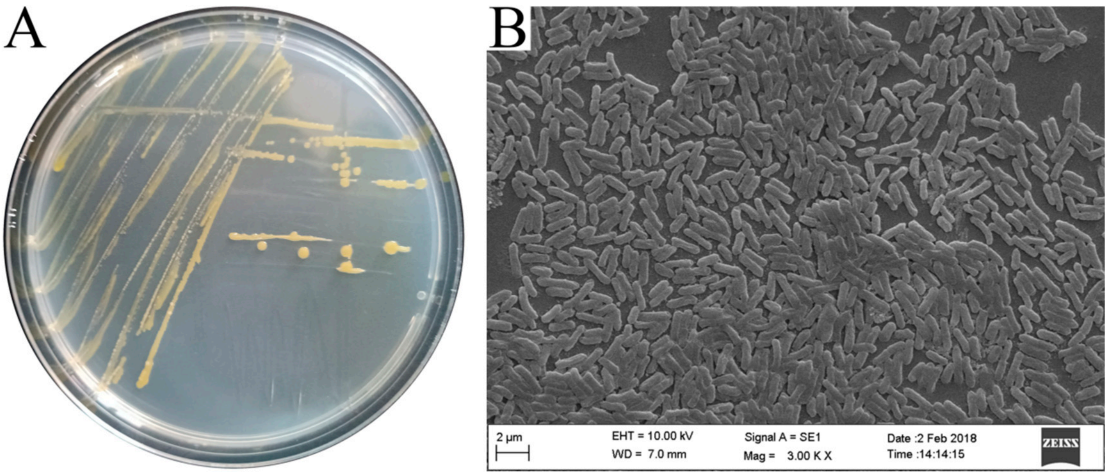

Figure S2 Morphological characterization of Pantoea alhagi NX-11(A) Colony morphology of NX-11. (B) Scanning electron micrograph of NX-11 colonies.

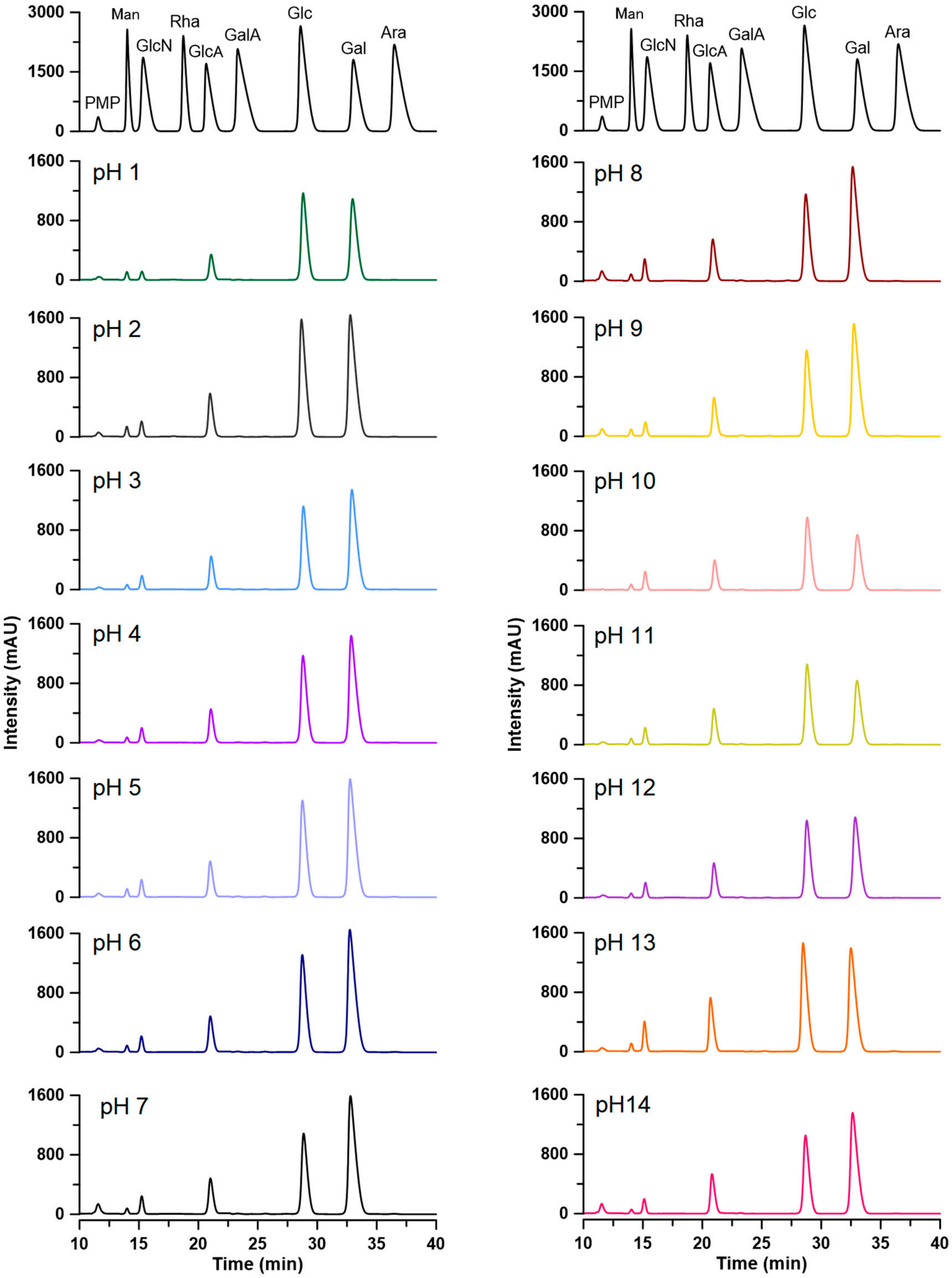

Figure S3. Analysis of monosaccharide components of pH 1-14-treated PAPS

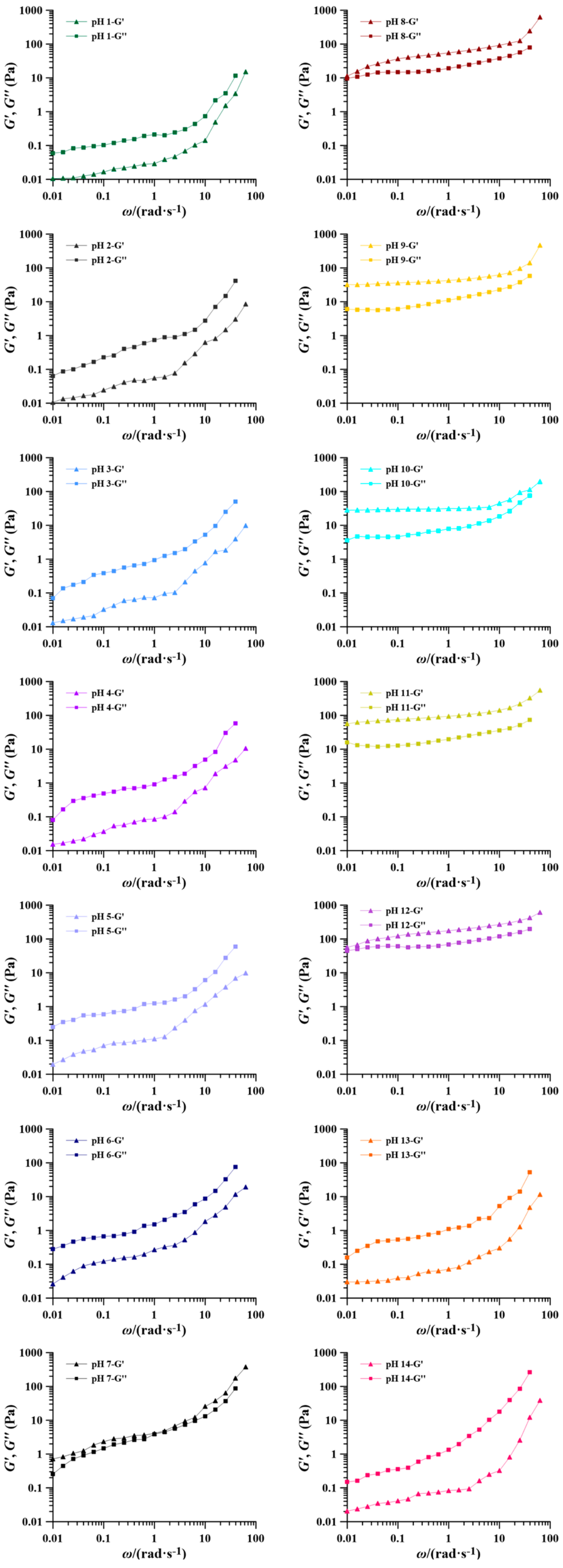

Figure. S4. Effect of pH on the storage (G') and loss (G'') moduli of pH 1-14-treated PAPS.
